# The IL-6 antagonist tocilizumab is associated with worse depression and related symptoms in the medically ill

**DOI:** 10.1038/s41398-020-01164-y

**Published:** 2021-01-18

**Authors:** Jennifer M. Knight, Erin S. Costanzo, Suraj Singh, Ziyan Yin, Aniko Szabo, Deepa S. Pawar, Cecilia J. Hillard, J. Douglas Rizzo, Anita D’Souza, Marcelo Pasquini, Christopher L. Coe, Michael R. Irwin, Charles L. Raison, William R. Drobyski

**Affiliations:** 1 Department of Psychiatry and Behavioral Medicine, Medical College of Wisconsin, Milwaukee, WI USA; 2 Section of BMT & Cellular Therapies; Division of Hematology/Oncology, Department of Medicine, Medical College of Wisconsin, Milwaukee, WI USA; 3 Department of Microbiology & Immunology, Medical College of Wisconsin, Milwaukee, WI USA; 4 Department of Psychiatry, University of Wisconsin School of Medicine and Public Health, Madison, WI USA; 5 Division of Biostatistics, Institute for Health & Society; Medical College of Wisconsin, Milwaukee, WI USA; 6 Department of Pharmacology and Toxicology and Neuroscience Research Center, Medical College of Wisconsin, Milwaukee, WI USA; 7 Center for International Blood and Marrow Transplant Research, Medical College of Wisconsin, Milwaukee, WI USA; 8 Department of Psychology, University of Wisconsin, Madison, WI USA; 9 Norman Cousins Center for Psychoneuroimmunology at the Jane and Terry Semel Institute for Neuroscience, and the Department of Psychiatry and Biobehavioral Sciences; David Geffen School of Medicine, University of California Los Angeles, Los Angeles, CA USA; 10 School of Human Ecology, University of Wisconsin-Madison, Madison, WI USA; 11 Usona Institute, Fitchburg, WI USA

**Keywords:** Depression, Human behaviour

## Abstract

Because medical illness is associated with increased inflammation and an increased risk for treatment-resistant major depressive disorder, anti-cytokine therapy may represent a novel, and especially efficacious, treatment for depression. We hypothesized that blockade of the interleukin (IL)-6 signaling pathway with tocilizumab would decrease depression and related symptomatology in a longitudinal cohort of allogeneic hematopoietic stem cell transplantation (HCT) patients, a medically ill population with a significant inflammation and psychopathology. Patients undergoing allogeneic HCT received either a single dose of tocilizumab one day prior to HCT (*n* = 25), or HCT alone (*n* = 62). The primary outcome included depressive symptoms at 28 days post HCT; anxiety, fatigue, sleep, and pain were assessed at pretreatment baseline and days +28, +100, and +180 post HCT as secondary outcomes. Multivariate regression demonstrated that preemptive treatment with tocilizumab was associated with significantly higher depression scores at D28 vs. the comparison group (*β* = 5.74; 95% CI 0.75, 10.73; *P* = 0.03). Even after adjustment for baseline depressive symptoms, propensity score, and presence of acute graft-versus-host disease (grades II–IV) and other baseline covariates, the tocilizumab-exposed group continued to have significantly higher depression scores compared to the nonexposed group at D28 (*β* = 4.73; 95% CI 0.64, 8.81; *P* = 0.02). Despite evidence that IL-6 antagonism would be beneficial, blockade of the IL-6 receptor with tocilizumab among medically ill patients resulted in significantly more—not less—depressive symptoms.

## Introduction

Major depressive disorder (MDD), with a lifetime prevalence of 20% (ref. ^1^), is even more common in the context of medical illness, with a prevalence rate twice that seen in the general population^2–4^. Depression in the medically ill is also significantly more likely to be treatment resistant^5^. Inflammation, a common feature of most medical illnesses, has been associated with specific patterns of brain dysfunction in patients with MDD^6^ and is thought to contribute to depression in medically ill and healthy individuals^7–9^.

Recent data suggest that blocking inflammatory signaling pathways with cytokine antagonists may provide a novel treatment approach for patients with MDD^10–13^. This may be especially true for those with a history of treatment resistance, particularly among those with high levels of inflammation, as occurs with medical comorbidities. Prior work has focused primarily on tumor necrosis factor (TNF) blockade^11,14^. Given the incomplete response of TNF blockade to treat depression^14^, interleukin (IL)-6 blockade may be an effective strategy^15,16^ since IL-6 is reliably elevated in MDD^17–22^ and associated with induction of depression following inflammatory challenge^13,14^.

Tocilizumab antagonizes IL-6 activation of both classical (membrane-bound) and trans (soluble) receptor signaling^15^. Two published studies evaluate associations between tocilizumab administration and MDD-related symptoms (depression, anxiety, and fatigue), with both concluding that tocilizumab produced beneficial effects. However, these studies had several limitations, including recruitment limited to patients with rheumatoid arthritis (whose symptoms improved with disease remission), small sample sizes^16^, and absence of a control group^16,23^.

Hematopoietic cell transplantation (HCT) patients constitute a medically ill cancer population with a significant degree of inflammation and depression in the peri-HCT period^24–26^. Depressive symptoms constitute a significant prognostic risk variable for poorer clinical outcomes among HCT recipients, including early mortality and increased occurrence of graft-versus-host disease (GVHD)^27,28^. IL-6 is dysregulated during GVHD^29–31^, with IL-6 antagonists demonstrating promise for GVHD prevention^32–35^. HCT patients are a clinically relevant population to address the larger question of whether IL-6 antagonism might offer a viable antidepressant strategy among medically ill patients experiencing elevated inflammatory activity.

We conducted an observational cohort study to evaluate the potentially beneficial effects of tocilizumab on depression and related symptoms among allogeneic HCT recipients, and compared their outcomes to a cohort of allogeneic HCT recipients who did not receive tocilizumab. The primary objective was to evaluate whether tocilizumab use was associated with less severe depressive symptoms following HCT. Secondary outcomes included the effects of tocilizumab treatment on anxiety, fatigue, sleep, and pain following HCT. The a priori prediction was that allogeneic HCT patients given tocilizumab would have less severe depression and related symptoms, as compared to those not given tocilizumab.

## Methods

### Study overview and eligibility criteria

#### Intervention cohort

In addition to the standard GVHD prophylaxis with tacrolimus/methotrexate, individuals participating in a phase II open label trial at the Medical College of Wisconsin (MCW) also received tocilizumab for prevention of acute GVHD (aGVHD) after allogeneic HCT (clinicaltrials.gov NCT02206035). They were invited to provide pre- and post-HCT patient-reported outcome (PRO) data as part of the tocilizumab treatment arm for the current study. Patients were enrolled from January 2015 through July 2016. Eligibility for the parent and current study included being 18–75 years of age, and undergoing HCT for acute leukemia, chronic myelogenous leukemia (CML), myelodysplasia, other myeloproliferative disorders, or chemotherapy sensitive lymphoproliferative diseases. Patients were excluded if they had a history of intolerance or allergy to tocilizumab, or if they received rituximab or other monoclonal antibodies during conditioning. See Drobyski et al.^36^ for additional information on the intervention population and treatment regimen. Twenty-five of the 35 patients involved in the parent trial consented to the study. Administration of tocilizumab occurred through the MCW HCT Program. All participants provided written informed consent; all procedures were approved in advance by the MCW Institutional Review Board (IRB).

#### Comparison group

Individuals participating in a longitudinal study evaluating biobehavioral effects on recovery following allogeneic HCT at the University of Wisconsin–Madison (UW) served as a comparison group. UW patients received standard GVHD prophylaxis with methotrexate/tacrolimus; none received tocilizumab. Allogeneic transplant participants (*N* = 204) with follow-up PROs were enrolled between 2008 and 2015. Patients from the UW cohort who were receiving a second transplant, BEAM conditioning, total body irradiation, anti-thymocyte globulin, sirolimus, cord blood transplant, or cyclosporine/mycophenolate mofetil GVHD prophylaxis were excluded to maintain the treatment homogeneity between comparative cohorts, as these regimens were not used within the MCW tocilizumab group. No inflammatory biomarker data was available from this cohort. All participants provided written informed consent and all procedures were approved by the UW IRB.

#### Patient-reported outcomes

All participants completed self-report surveys at four time points: baseline (prior to tocilizumab for intervention group), day +28 (D28; with respect to day 0 being day of transplant), D100, and D180 post transplant. To assess the primary study endpoint (depression score at D28), the 20-item General Depression subscale from the Inventory of Depression and Anxiety Symptoms (IDAS) was used^37^. Questionnaires administered at all assessments included the IDAS, Fatigue Symptom Inventory (FSI; fatigue)^38^, Pittsburgh Sleep Quality Index (PSQI; sleep)^39^, and Brief Pain Inventory (pain)^40^. Patients endorsing thoughts of suicidality or self-harm per the IDAS were contacted by the study principal investigator and offered appropriate follow-up care.

### Sample size

The study sample size was determined by the parent studies, which were designed for outcomes unrelated to the primary aim of the current analyses. With the final sample size of 25 tocilizumab-treated and 62 comparison patients, the study has 80% power to detect effects of 0.67 standard deviations (SD) or higher, and 90% power to detect effects of 0.78 SD. Previously published articles report effect sizes ranging between 0.62 and 1.25 on the General Depression subscale of the IDAS between cases and non-cases for a variety of psychiatric disorders^37^. Assuming a SD of 12 points, an 8-point difference in the IDAS depression scale was detectable with at least 80% power.

### Statistical analysis

#### Descriptive analyses

Student’s *t* test or Chi-square tests were used to evaluate differences between the two cohorts for continuous or categorical baseline demographic variables, respectively. Univariate comparisons of PRO variables were performed using Wilcoxon’s rank-sum test. Demographic characteristics described for each group include age, sex, race, body mass index (BMI), income, and education level. Additional medical characteristics for each group include disease, cytomegalovirus (CMV) status, conditioning regimen intensity, donor and HLA match, and graft type.

#### Primary analysis

An analysis of covariance (ANCOVA) regression model was fitted for all eligible patients to evaluate the impact of cohort (tocilizumab vs. comparison group) on the primary outcome of depressive symptoms at D28, while covarying for both baseline depressive symptomatology and presence of grades II–IV aGVHD by D28.

#### Secondary analyses

Inverse propensity-weighted regression was the primary analytical approach for multivariable analyses of the trajectory of depressive symptoms over all time points within a single model. The trajectories of other PROs were analyzed similarly. Propensity scores were obtained using a logistic regression model with age, sex, BMI, CMV status, conditioning regimen, and donor type as predictors of study group. Graft source was unbalanced but could not be included in the propensity model due to the very low number of patients with bone marrow as graft source in the comparison group. For each PRO, a linear mixed-effect model was fitted with inverse propensity scores as weights to evaluate the impact of treatment group at each time point (D28, D100, and D180), adjusting for the baseline value of the measurement and grades II–IV aGVHD before the corresponding time point. For all tests, alpha level was set at two-sided *p* < 0.05, and no adjustments for multiple comparisons were performed.

#### Post hoc analyses

Because aGVHD status was included as a time-varying covariate in the multivariable analyses, the same model was used to evaluate whether development of aGVHD affected PRO responses following tocilizumab treatment. To examine the degree to which differences in disease state composition between the two study sites might have contributed to the findings, additional sensitivity analyses were conducted in a group that restricted disease states between the sites by removing disease subsets that had only 0 or 1 case in one of the groups. These analyses were limited to patients with acute myelogenous leukemia, acute lymphocytic leukemia, CML, and myelodysplastic syndrome (*n* = 25, tocilizumab; *n* = 29, comparison group).

In all analyses, the assumptions of the parametric tests (*t* test for age and regression models for the PRO outcomes) were evaluated using residual plots, including QQ plots to assess normality and residual vs. fitted value plots for homoscedasticity.

## Results

### Descriptive analyses

**Fig. 1:**
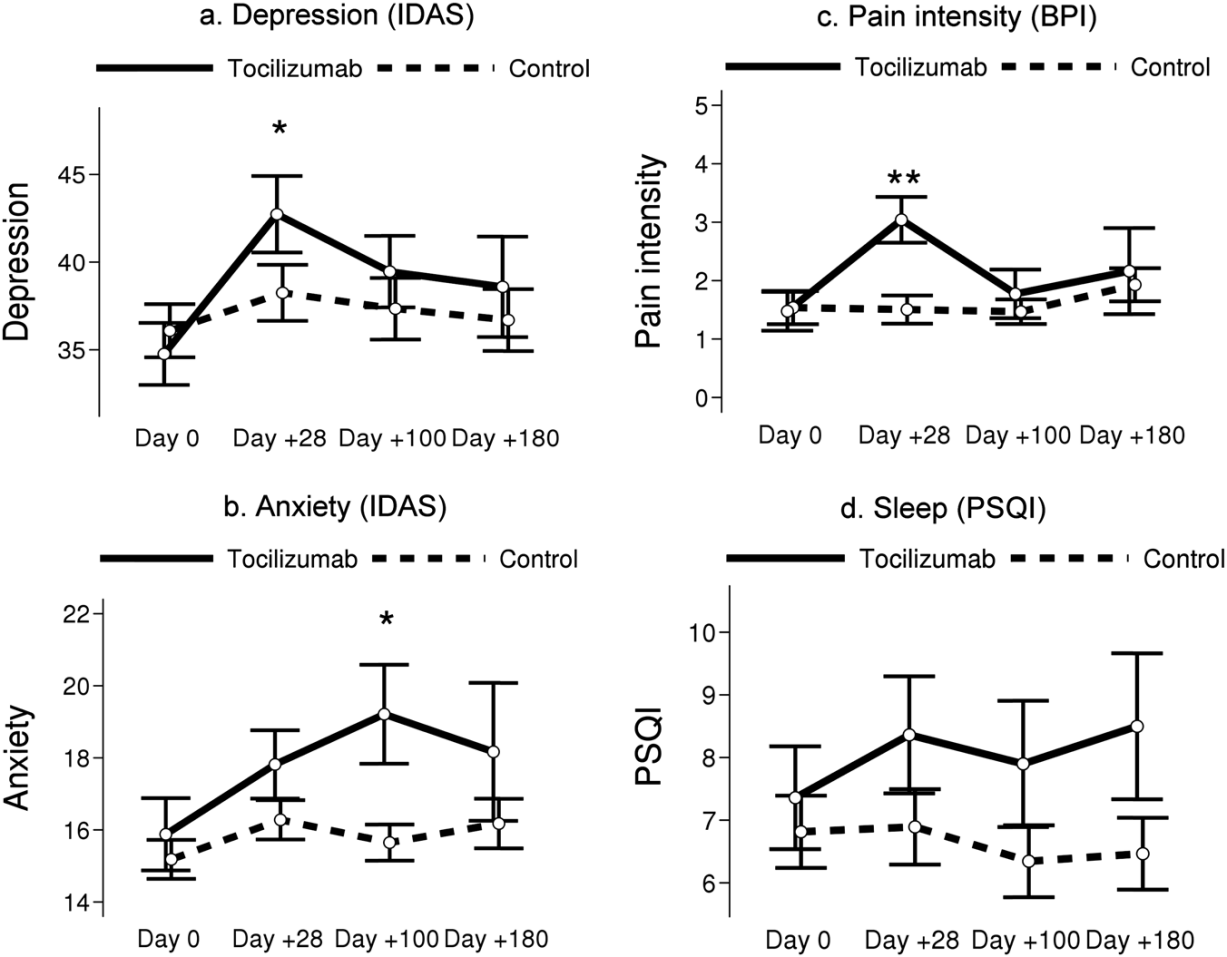
Mean patient-reported outcome scores by study group and time. Error bars represent standard errors. Significance stars are based on ANCOVA after adjusting for baseline depression (*P = 0.01–0.05, **P < 0.01).

Baseline demographic characteristics are summarized in Table 1. Age was the only demographic variable differing between the cohorts, with the tocilizumab cohort slightly older than the comparison group (60.2 vs. 53.9, *P* = 0.01). The tocilizumab group had a higher proportion of patients receiving a bone marrow graft (20% vs. 5%, *P* = 0.03). There were no significant differences in sleep (Fig. 1d) or development of aGVHD (Table 1), although the tocilizumab group had consistently worse PSQI scores and a lower incidence of aGVHD (for further details on parent study outcomes, see Drobyski et al.^36^).

**Table 1:** Demographic and clinical characteristics.

Characteristic	Participants, no. (%)		
	Tocilizumab cohort (*N* = 25)	Control cohort (*N* = 62)	*P* value
Age, mean (SD), y	60.2 (10.6)	53.9 (10.1)	0.01
Female sex	10 (40)	21 (34)	0.59
Race			0.14
Caucasian/White	23 (92)	61 (98)	
Asian American	1 (4)	0 (0)	
Latino/a	1 (4)	0 (0)	
Native American	0 (0)	1 (2)	
Body mass index, mean (SD)	28.4 (4.6)	28.8 (4.9)	0.74
Education^a,b^			0.90
<12 years	1 (4)	3 (5)	
High school	8 (33)	20 (33)	
Some college	5 (21)	13 (21)	
College graduate	4 (17)	14 (23)	
Post graduate degree	5 (21)	7 (12)	
Trade school	1 (4)	4 (7)	
Income^c,d^			
≤$10,000	0 (0)	1 (2)	0.76
$10,001–$25,000	4 (17)	6 (10)	
$25,001–$40,000	4 (17)	15 (25)	
$40,001–$55,000	4 (17)	6 (10)	
$55,001–$70,000	3 (13)	8 (14)	
$70,001–$85,000	3 (13)	6 (10)	
$85,001–$100,000	1 (4)	9 (15)	
>$100,000	4 (17)	8 (14)	
Diagnostic category			
ALL	2 (8)	2 (3)	<0.001
AML	15 (60)	17 (27)	
CLL	0 (0)	9 (15)	
CML	5 (20)	2 (3)	
Lymphoma	1 (4)	22 (35)	
MDS	2 (8)	8 (13)	
Other	0 (0)	2 (3)	
Donor type			
8/8 HLA matched unrelated donor	14 (56)	24 (39)	0.14
HLA matched sibling donor	11 (44)	38 (61)	
Graft type^c^			
Bone marrow	5 (20)	3 (5)	0.03
Mobilized peripheral blood stem cells	20 (80)	56 (95)	
CMV positive^a^	9 (36)	32 (53)	0.17
Conditioning regimen			
Myeloablative	12 (48)	27 (44)	0.71
Reduced intensity conditioning	13 (52)	35 (57)	
Development of aGVHD (grades II–IV)			
Baseline	0 (0)	0 (0)	
Day 28	0 (0)	3 (5)	0.26
Day 100	2 (8)	10 (16)	0.32
Day 180	3 (12)	18 (29)	0.09

*ALL* acute lymphoblastic leukemia, *AML* acute myelogenous leukemia, *CLL* chronic lymphocytic leukemia, *CML* chronic myelogenous leukemia, *CMV* cytomegalovirus, *HLA* human leukocyte antigen, *MDS* myelodysplasia.

^a^Data missing for one participant in the control cohort.

^b^Data missing for one participant in the tocilizumab cohort.

^c^Data missing for three participants in the control cohort.

^d^Data missing for two participants in the tocilizumab cohort.

Univariate PRO characteristics for the entire cohort (*n* = 87) are reported in Table 2. Depressive symptoms were significantly worse at D28 (42.7 vs. 38.2, *P* = 0.03, Fig. 1a) and anxiety significantly higher at D100 (19.2 vs. 15.6, *P* = 0.02, Fig. 1b) among patients receiving tocilizumab compared to those not receiving the drug. While fatigue intensity was significantly lower (2.5 vs. 3.9, *P* = 0.003) among the tocilizumab patients at baseline, fatigue intensity was higher among this cohort at three subsequent time points, although this trend did not attain statistical significance. Patients receiving tocilizumab reported significantly more pain intensity at D28 than those not receiving tocilizumab (3.0 vs.1.5, *P* < .001, Fig. 1c).

**Table 2:** Quality of life patient-reported outcomes at baseline, day 28, day 100, and day 180 post transplant.

Assessment	All (*N* = 87)		Tocilizumab cohort (*N* = 25)		Control cohort (*N* = 62)		
	No. of participants	Assessment data, mean score (SD)	No. of participants	Assessment data, mean score (SD)	No. of participants	Assessment data, mean score (SD)	*P* value
Depression							
Baseline	85	35.7 (10.9)	25	34.8 (8.8)	60	36.1 (11.7)	0.95
Day 28	79	39.5 (11.7)	22	42.7 (10.2)	57	38.2 (12.1)	0.03
Day 100	74	37.9 (12.0)	20	39.5 (9.1)	54	37.3 (12.9)	0.16
Day 180	57	37.1 (11.4)	12	38.6 (9.9)	45	36.7 (11.8)	0.40
Anxiety							
Baseline	85	15.4 (4.4)	25	15.9 (5.0)	60	15.2 (4.2)	0.66
Day 28	79	16.7 (4.2)	22	17.8 (4.4)	57	16.3 (4.1)	0.10
Day 100	73	16.6 (4.6)	19	19.2 (6.0)	54	15.6 (3.7)	0.02
Day 180	57	16.6 (5.1)	12	18.2 (6.6)	45	16.2 (4.6)	0.44
Fatigue, intensity							
Baseline	85	3.4 (2.1)	25	2.5 (1.5)	60	3.9 (2.2)	0.003
Day 28	79	4.0 (1.8)	22	4.2 (1.7)	57	3.9 (1.9)	0.36
Day 100	73	3.4 (2.0)	18	3.7 (2.0)	55	3.3 (2.0)	0.52
Day 180	55	3.7 (1.8)	12	4.2 (1.1)	43	3.6 (2.0)	0.36
Fatigue, duration							
Baseline	85	6.5 (4.5)	25	5.4 (4.1)	60	7.0 (4.6)	0.17
Day 28	79	8.8 (4.5)	22	9.6 (4.7)	57	8.5 (4.4)	0.28
Day 100	74	8.0 (4.5)	19	8.9 (4.4)	55	7.7 (4.6)	0.31
Day 180	55	8.3 (4.5)	12	9.2 (4.0)	43	8.1 (4.6)	0.61
Fatigue, interference							
Baseline	85	2.2 (2.3)	25	1.5 (1.8)	60	2.5 (2.4)	0.09
Day 28	79	2.9 (2.4)	22	2.8 (1.9)	57	2.9 (2.5)	0.79
Day 100	74	2.4 (2.3)	19	2.3 (1.8)	55	2.5 (2.5)	0.83
Day 180	55	2.3 (2.1)	12	1.9 (1.7)	43	2.5 (2.2)	0.53
Pain, intensity							
Baseline	84	1.5 (2.0)	25	1.5 (1.7)	59	1.5 (2.2)	0.50
Day 28	79	1.9 (1.9)	22	3.0 (1.8)	57	1.5 (1.8)	<.001
Day 100	73	1.5 (1.6)	18	1.8 (1.8)	55	1.5 (1.6)	0.61
Day 180	57	2.0 (2.0)	12	2.2 (2.6)	45	1.9 (1.9)	0.93
Pain, interference							
Baseline	84	1.3 (2.0)	25	0.9 (1.7)	59	1.4 (2.1)	0.22
Day 28	78	1.6 (2.2)	22	2.0 (2.0)	56	1.5 (2.4)	0.08
Day 100	73	1.6 (2.3)	18	1.6 (1.8)	55	1.6 (2.4)	0.64
Day 180	57	2.0 (2.4)	12	1.6 (2.4)	45	2.1 (2.4)	0.42
Sleep							
Baseline	85	7.0 (4.3)	25	7.4 (4.1)	60	6.8 (4.5)	0.44
Day 28	79	7.3 (4.5)	22	8.4 (4.4)	57	6.9 (4.5)	0.12
Day 100	75	6.8 (4.3)	20	7.9 (4.5)	55	6.3 (4.3)	0.16
Day 180	57	6.9 (3.9)	12	8.5 (4.0)	45	6.5 (3.8)	0.13

### Primary analysis

ANCOVA adjusting for baseline depressive symptoms and presence of grades II–IV aGVHD demonstrated that the tocilizumab group had significantly higher depression scores at D28 vs. the comparison group (Fig. 1; *β* = 5.74; 95% CI 0.75, 10.73; *P* = 0.03).

### Secondary analyses

After adjusting for baseline depressive symptoms, presence of aGVHD (grades II–IV), and additional baseline covariates via propensity weighting, the tocilizumab-exposed group continued to have significantly higher depression scores at D28 compared to the unexposed group (*β* = 4.73; 95% CI 0.64, 8.81; *P* = 0.02), a difference that resolved and was no longer significant at D100 and D180 (Table 3). Patients receiving tocilizumab exhibited significantly more severe anxiety symptoms (*β* = 2.82; 95% CI 0.86, 4.78; *P* = 0.005) at D100 (with propensity weighting after adjusting for baseline anxiety and presence of aGVHD; Table 3). None of the FSI variables were significantly different between the treatment groups at any time point. Adjusted models for pain intensity indicated that there were significantly more pain symptoms in the tocilizumab-treated vs. unexposed patients at D28 (Table 3; *β* = 1.22; 95% CI 0.49, 1.96; *P* = 0.001) and D180 (Table 3; *β* = 0.98; 95% CI 0.07, 1.88; *P* = 0.04). Finally, the tocilizumab-exposed group reported significantly poorer sleep quality at D180 (Table 3; *β* = 2.46; 95% CI 0.54, 4.38; *P* = 0.01).

**Table 3:** Tocilizumab compared to control cohort based on propensity-weighted^a^ models for quality of life patient-reported outcomes after adjusting for baseline values and aGVHD (grades II–IV).

Assessment	Day 28		Day 100		Day 180	
	Estimate (95% CI)	*P* value	Estimate (95% CI)	*P* value	Estimate (95% CI)	*P* value
Depression	4.73 (0.64, 8.81)	0.02	1.54 (−2.81, 5.88)	0.49	4.41 (−0.59, 9.40)	0.08
Anxiety	1.64 (−0.16, 3.44)	0.07	2.82 (0.86, 4.78)	0.005	1.76 (−0.48, 4.00)	0.12
Fatigue, intensity	0.36 (−0.41, 1.13)	0.36	0.18 (−0.66, 1.01)	0.68	0.89 (−0.05, 1.82)	0.06
Fatigue, duration	1.13 (−0.56, 2.83)	0.19	1.11 (−0.70, 2.93)	0.23	1.22 (−0.85, 3.30)	0.25
Fatigue, interference	−0.31 (−1.16, 0.54)	0.47	−0.27 (−1.18, 0.64)	0.56	−0.37 (−1.41, 0.67)	0.48
Pain, intensity	1.22 (0.49, 1.96)	0.001	0.27 (−0.53, 1.07)	0.50	0.98 (0.07, 1.88)	0.04
Pain, interference	0.11 (−0.78, 0.99)	0.81	−0.15 (−1.13, 0.82)	0.75	0.08 (−1.02, 1.19)	0.88
Sleep	1.32 (−0.30, 2.94)	0.11	1.47 (−0.23, 3.17)	0.09	2.46 (0.54, 4.38)	0.01

^a^Propensity scores were obtained using a logistic regression model with age, sex, BMI, CMV status, conditioning regimen, and donor type as predictors of study group.

### Post hoc analyses

Among the measures acquired after progression to grades II–IV aGVHD in either the intervention or comparison group from any of the 187 data points collected (*n* = 22 data points for patients with aGVHD, or 11.8%), there was no significant difference in endorsement of depression (*β* = 3.13; 95% CI −1.77, 8.04; *P* = 0.19) or anxiety (*β* = 0.94; 95% CI −1.20, 3.08; *P* = 0.35) among those with aGVHD (Table 4). After developing aGVHD, patients experienced a significantly greater duration of fatigue (*β* = 2.56; 95% CI 0.36, 4.76; *P* = 0.03) compared to patients who did not develop aGVHD. Neither pain nor sleep quality was affected by development of aGVHD. The effect sizes for the secondary analyses that focused on the disease-restricted subset were similar to the full cohort (see Supplemental Tables 1–4 for additional details), indicating that any potential differences between the two study sites or disease state composition within did not substantially contribute to the primary findings.

**Table 4:** Effect of presence of aGVHD (grades II–IV) compared to those without aGVHD at same time point, based on propensity-weighted^a^ models for quality of life patient-reported outcomes after adjusting for baseline values and cohort.

Assessment	Estimate (95% CI)	*P* value
Depression	3.13 (−1.77, 8.04)	0.19
Anxiety	0.94 (−1.20, 3.08)	0.35
Fatigue, intensity	0.37 (−0.63, 1.36)	0.43
Fatigue, duration	2.56 (0.36, 4.76)	0.03
Fatigue, interference	1.01 (−0.08, 2.10)	0.07
Pain, intensity	0.25 (−0.66, 1.17)	0.55
Pain, interference	0.12 (−0.95, 1.19)	0.81
Sleep	−0.06 (−1.98, 1.87)	0.95

^a^Propensity scores were obtained using a logistic regression model with age, sex, BMI, CMV status, conditioning regimen, and donor type as predictors of study group.

## Discussion

Despite the prevailing psychiatric gestalt, based largely on studies examining TNF antagonists^12,13,41^, and contrary to our initial hypothesis, allogeneic HCT patients receiving the IL-6 antagonist tocilizumab experienced significantly worse—not better—depression, anxiety, pain, and sleep compared to HCT patients not receiving the drug. Antagonizing IL-6-mediated inflammation was not only ineffective at preventing adverse PROs, it was associated with worse symptomatology. Along with other emerging findings^42^, these data challenge the current psychiatric paradigm purporting that anti-cytokine therapy may be effective at mitigating depression symptoms, particularly among treatment-resistant or inflamed individuals. Importantly, the difference in depressive symptoms between groups in this study was clinically meaningful^43^. Further, findings from the current study have clinical implications for treatment-related QOL and QOL in the medically ill, further informing our understanding of biological processes underlying the basis for depression and related sickness symptoms in this population.

Inflammatory cytokines contribute to depressive symptoms, with antidepressant therapies effective at blocking this effect on the brain^44^. Meta-analyses suggest that anti-inflammatory treatments, including anti-cytokine therapies, mitigate depressive symptomatology^10,12^. A recent report of the antidepressant effects of anti-cytokine therapies indicates a potential causative role of cytokines in depression and a potential treatment role for cytokine modulators as novel drugs for depression in chronically inflamed individuals^12^. In that analysis, those treated with tocilizumab demonstrated statistically significant improvement in depressive symptoms^12^; however, this meta-analytic assessment was limited by several factors, including the existence of only two studies assessing the effects of tocilizumab on PRO measures^16,23^. The first prospective, randomized controlled trial of anti-cytokine therapy (infliximab) for depression treatment identified that TNF antagonism was effective at improving depressive symptoms only in patients with high inflammation^11^, although these results recently failed to be replicated in a cohort of bipolar patients^42^. Were this a uniform anti-cytokine therapy response, similar beneficial effects would be expected in the current study as the tocilizumab group had markedly elevated IL-6 levels, up to a ninefold increase from baseline^36^.

Given this, one must consider other possible immunobiologic mechanisms to explain the current findings. First, while tocilizumab is not believed to cross the blood–brain barrier^45^, emerging evidence suggests there is likely increased blood–brain permeability under conditions of significant inflammation, like HCT^46,47^. It is unknown if, or how, this would affect anti-cytokine signaling. Second, despite evidence that TNF antagonism reduces depressive symptoms among inflamed and depressed individuals^11^, it has alternatively been suggested that blanket blockade of anti-cytokine signaling may be inadvisable^48,49^. Finally, it is possible that blockade of peripheral receptors alone results in excess unbound peripheral IL-6 available to exert its action centrally. In the parent trial, tocilizumab-treated patients demonstrated a marked increase in IL-6 and sIL-6R at all time points when compared to control patients^36^. This notion is supported by data with the monoclonal IL-6 antibodies sirukumab and siltuximab, which target IL-6 itself and demonstrate efficacy over placebo to improve depressive symptoms among individuals with autoimmune disorders^50^.

Alternatively, it is possible that tocilizumab negates beneficial effects of IL-6, as data also suggest IL-6 has positive health associations. For example, acute exercise increases IL-6 100-fold without activating TNF or IL-1 beta, resulting in a constellation of biological changes generally considered anti-inflammatory^51^. Hyperthermia^52^ and fasting^53^ induce similar IL-6-increasing and health-promoting changes. Further, these studies generally support antidepressant or mood-enhancing properties as well, although these interventions only transiently increase IL-6. It is possible that other signaling molecules accompanying IL-6 are responsible for the current tocilizumab findings. For example, administration of IL-6 alone does not produce sickness behavior, but requires the presence of IL-1 to achieve this effect^54^. Finally, the anti-inflammatory cascade may be just one component of immunomodulation in the inflammation–depression relationship^48^. This notion is in part supported by data from our group identifying that IL-6 antagonism in mice with GVHD does not alter dysregulated brain tryptophan metabolism^33^, a monoamine pathway altered by neuroinflammation^6^.

Underscoring the clinical relevance of the current findings, tocilizumab received FDA approval for cytokine release syndrome (CRS) in 2017. CRS is a severe and common side effect of chimeric antigen receptor (CAR) T-cell therapy, a novel immunotherapy utilizing genetically modified T cells to specifically target cancer cells^55^. Inhibition of IL-6 binding with tocilizumab results in a rapid and dramatic improvement in all the life-threatening symptoms associated with CAR T therapies except for the neurologic sequelae^56^. Investigators postulate neurotoxicity may actually worsen with tocilizumab administration^57^, a finding in accord with the current data. However, blocking IL-1 alone abrogates the neurotoxic effects of CRS, further substantiating the idea that other inflammatory cytokines in addition to IL-6 might have a role in promoting neurotoxicity^58^. Finally, tocilizumab is being increasingly used to treat patients with severe COVID-19 who have CRS^59^, rendering it particularly timely to understand its neuropsychiatric sequelae^60^.

This study’s findings are limited in several respects. Most notably, the intervention vs. comparison cohorts were treated at different institutions. While this may have confounded study findings, several factors argue against this. First, the tocilizumab cohort endorsed better QOL symptoms upon study initiation yet developed more severe symptomatology following tocilizumab and HCT. Second, tocilizumab’s impact on aGVHD should have biased the data in the opposite direction of the current findings. In fact, while patients with aGVHD were more depressed than patients without, the tocilizumab group remained overall more depressed, despite having less aGVHD. Third, the post hoc comparison group received significantly more myeloablative conditioning regimens—a more pro-inflammatory and toxic treatment—than the tocilizumab group. Again, this group difference should have biased the data in the opposite direction of the current findings. Fourth, the current results are consistent across PRO domains and time points, rendering it possible, but unlikely that institutionally related factors influenced these particular outcomes with such uniformity. Another limitation is that the tocilizumab group was older than the control group. In sum, future research should target intervention and control populations from the same institution using a randomized, blinded, parallel group design. Finally, while cytokine data was available and reported by Drobyski et al.^36^ for the tocilizumab group, inflammatory biomarker data from the comparison cohort was not available. Of note, both groups had generally modest levels of depression symptomatology. Therefore, results may not generalize to moderately or severely depressed populations who are in need of novel treatment approaches. Future studies should compare biological parameters of inflammation—including but not limited to IL-6—between treatment vs. control conditions to better understand the physiologic milieu underlying the observed clinical differences. While the current study did not have access to participant biological data in the comparison group, such inflammatory biology data collection is currently underway to better understand tocilizumab-associated inflammatory signaling, and its relationship to PROs and symptomatology.

The current findings suggest that the IL-6 receptor antagonist tocilizumab does not improve and may actually worsen depression and related symptoms—including anxiety, pain, and sleep—among medically ill individuals undergoing allogeneic HCT. These are the first human data evaluating both an intervention and comparison group in the setting of tocilizumab administration. Given prior dogma supporting the beneficial effects of anti-cytokine therapy for inflammation-associated mood dysregulation, these findings have clinical implications both in informing current psychiatric paradigm, as well as anticipating potential adverse QOL and psychiatric sequelae among patients receiving anti-cytokine therapy for medical purposes.

## Supplementary information

Supplemental Tables for Disease-Restricted Subset Analysis

## References

[1] Kessler RC (2003). The epidemiology of major depressive disorder: results from the National Comorbidity Survey Replication (NCS-R). JAMA.

[2] Garrido MM (2017). Mental illness and mental healthcare receipt among hospitalized veterans with serious physical illnesses. J. Palliat. Med..

[3] Mikocka-Walus A, Knowles SR, Keefer L, Graff L (2016). Controversies revisited: a systematic review of the comorbidity of depression and anxiety with inflammatory bowel diseases. Inflamm. Bowel Dis..

[4] Derogatis LR (1983). The prevalence of psychiatric disorders among cancer patients. JAMA.

[5] Popkin MK, Callies AL, Mackenzie TB (1985). The outcome of antidepressant use in the medically ill. Arch. Gen. Psychiatry.

[6] Felger JC, Lotrich FE (2013). Inflammatory cytokines in depression: neurobiological mechanisms and therapeutic implications. Neuroscience.

[7] Miller AH, Maletic V, Raison CL (2009). Inflammation and its discontents: the role of cytokines in the pathophysiology of major depression. Biol. Psychiatry.

[8] Lanquillon S, Krieg J-C, Bening-Abu-Shach U, Vedder H (2000). Cytokine production and treatment response in major depressive disorder. Neuropsychopharmacology.

[9] Misiak B (2018). Cytokine alterations and cognitive impairment in major depressive disorder: from putative mechanisms to novel treatment targets. Prog. Neuropsychopharmacol. Biol. Psychiatry.

[10] Köhler O (2014). Effect of anti-inflammatory treatment on depression, depressive symptoms, and adverse effects: a systematic review and meta-analysis of randomized clinical trials. JAMA Psychiatry.

[11] Raison CL (2013). A randomized controlled trial of the tumor necrosis factor antagonist infliximab for treatment-resistant depression: the role of baseline inflammatory biomarkers. JAMA Psychiatry.

[12] Kappelmann N, Lewis G, Dantzer R, Jones PB, Khandaker GM (2016). Antidepressant activity of anti-cytokine treatment: a systematic review and meta-analysis of clinical trials of chronic inflammatory conditions. Mol. Psychiatry.

[13] Udina M (2014). Cytokine-induced depression: current status and novel targets for depression therapy. CNS Neurol. Disord. Drug Targets.

[14] Roman M, Irwin MR (2019). Novel neuroimmunologic therapeutics in depression: a clinical perspective on what we know so far. Brain Behav. Immun..

[15] Scheller J, Rose-John S (2006). Interleukin-6 and its receptor: from bench to bedside. Med. Microbiol. Immunol..

[16] Traki L (2014). Responsiveness of the EuroQol EQ-5D and Hospital Anxiety and Depression Scale (HADS) in rheumatoid arthritis patients receiving tocilizumab. Clin. Rheumatol..

[17] Khandaker GM, Pearson RM, Zammit S, Lewis G, Jones PB (2014). Association of serum interleukin 6 and C-reactive protein in childhood with depression and psychosis in young adult life: a population-based longitudinal study. JAMA Psychiatry.

[18] Stewart JC, Rand KL, Muldoon MF, Kamarck TW (2009). A prospective evaluation of the directionality of the depression-inflammation relationship. Brain, Behav. Immun..

[19] Dowlati Y (2010). A meta-analysis of cytokines in major depression. Biol. Psychiatry.

[20] Alesci S (2005). Major depression is associated with significant diurnal elevations in plasma interleukin-6 levels, a shift of its circadian rhythm, and loss of physiological complexity in its secretion: clinical implications. J. Clin. Endocrinol. Metab..

[21] El-Gohary GM, Azzam HM, Ahmed OI, El-Shokry MH (2008). Pro-inflammatory cytokines and depression in patients with acute leukemia. Egypt. J. Immunol..

[22] Miller GE, Cole SW (2012). Clustering of depression and inflammation in adolescents previously exposed to childhood adversity. Biol. Psychiatry.

[23] Gossec L, Steinberg G, Rouanet S, Combe B (2015). Fatigue in rheumatoid arthritis: quantitative findings on the efficacy of tocilizumab and on factors associated with fatigue. The French multicentre prospective PEPS Study. Clin. Exp. Rheumatol..

[24] Knight JM, Lyness JM, Sahler OJZ, Liesveld JL, Moynihan JA (2013). Psychosocial factors and hematopoietic stem cell transplantation: potential biobehavioral pathways. Psychoneuroendocrinology.

[25] Costanzo ES, Juckett MB, Coe CL (2013). Biobehavioral influences on recovery following hematopoietic stem cell transplantation. Brain Behav. Immun..

[26] McQuellon RP (1998). Quality of life and psychological distress of bone marrow transplant recipients: the ‘time trajectory’ to recovery over the first year. Bone Marrow Transplant..

[27] Loberiza FR (2002). Association of depressive syndrome and early deaths among patients after stem-cell transplantation for malignant diseases. J. Clin. Oncol..

[28] El-Jawahri A (2017). Impact of pre-transplant depression on outcomes of allogeneic and autologous hematopoietic stem cell transplantation. Cancer.

[29] Hill GR, Krenger W, Ferrara JL (1997). The role of cytokines in acute graft-versus-host disease. Cytokines Cell. Mol. Ther..

[30] MacDonald KP, Hill GR, Blazar BR (2017). Chronic graft-versus-host disease: biological insights from preclinical and clinical studies. J. Am. Soc. Hematol..

[31] Zeiser R, Blazar BR (2017). Acute graft-versus-host disease—biologic process, prevention, and therapy. N. Engl. J. Med..

[32] Abboud R (2016). Severe cytokine-release syndrome after T cell–replete peripheral blood Haploidentical donor transplantation is associated with poor survival and anti–IL-6 therapy is safe and well tolerated. Biol. Blood Marrow Transplant..

[33] Belle L (2017). Host interleukin 6 production regulates inflammation but not tryptophan metabolism in the brain during murine GVHD. Host interleukin 6 production regulates inflammation but not tryptophan metabolism in the brain during murine GVHD. JCI Insight.

[34] Beebe KL (2018). Tocilizumab in the treatment of pediatric chronic Gvhd. Biol. Blood Marrow Transplant..

[35] Kennedy GA (2014). Addition of interleukin-6 inhibition with tocilizumab to standard graft-versus-host disease prophylaxis after allogeneic stem-cell transplantation: a phase 1/2 trial. Lancet Oncol..

[36] Drobyski WR (2018). Tocilizumab, tacrolimus and methotrexate for the prevention of acute graft versus host disease: low incidence of lower gastrointestinal tract disease. Haematologica.

[37] Watson D (2007). Development and validation of the Inventory of Depression and Anxiety Symptoms (IDAS). Psychol. Assess..

[38] Hann DM (1998). Measurement of fatigue in cancer patients: development and validation of the Fatigue Symptom Inventory. Qual. Life Res..

[39] Vargas S (2010). Sleep dysfunction and psychosocial adaptation among women undergoing treatment for non-metastatic breast cancer. Psycho-Oncol..

[40] Cleeland, C. S. In *Effect of Cancer on Quality of Life* (ed. Osoba, D.) 293–305 (CRC Press, Boca Raton, FL, 1991).

[41] Brietzke E, Scheinberg M, Lafer B (2011). Therapeutic potential of interleukin-6 antagonism in bipolar disorder. Med. Hypotheses.

[42] McIntyre RS (2019). Efficacy of adjunctive infliximab vs placebo in the treatment of adults with bipolar I/II depression: a randomized clinical trial. JAMA Psychiatry.

[43] Stasik-O’Brien SM (2019). Clinical utility of the Inventory of Depression and Anxiety Symptoms (IDAS). Assessment.

[44] Hannestad J, DellaGioia N, Bloch M (2011). The effect of antidepressant medication treatment on serum levels of inflammatory cytokines: a meta-analysis. Neuropsychopharmacology.

[45] Nellan A (2018). Improved CNS exposure to tocilizumab after cerebrospinal fluid compared to intravenous administration in rhesus macaques. Blood.

[46] Abbott NJ (2000). Inflammatory mediators and modulation of blood–brain barrier permeability. Cell. Mol. Neurobiol..

[47] Ryu JK, McLarnon JG (2009). A leaky blood–brain barrier, fibrinogen infiltration and microglial reactivity in inflamed Alzheimer’s disease brain. J. Cell. Mol. Med..

[48] Eyre HA, Baune BT (2015). Anti-inflammatory intervention in depression. JAMA Psychiatry.

[49] Eyre H, Baune BT (2012). Neuroplastic changes in depression: a role for the immune system. Psychoneuroendocrinology.

[50] Sun Y (2017). The effects of interleukin-6 neutralizing antibodies on symptoms of depressed mood and anhedonia in patients with rheumatoid arthritis and multicentric Castleman’s disease. Brain Behav. Immun..

[51] Pedersen BK, Febbraio MA (2008). Muscle as an endocrine organ: focus on muscle-derived interleukin-6. Physiol. Rev..

[52] Raison C (2017). Inflammation in treatment resistant depression: challenges and opportunities. Biol. Psychiatry.

[53] Wueest S (2014). Interleukin-6 contributes to early fasting-induced free fatty acid mobilization in mice. Am. J. Physiol. Regul. Integr. Comp. Physiol..

[54] Lenczowski M (1999). Central administration of rat IL-6 induces HPA activation and fever but not sickness behavior in rats. Am. J. Physiol. Regul. Integr. Comp. Physiol..

[55] Le, R. Q. et al. FDA approval summary: tocilizumab for treatment of chimeric antigen receptor T cell‐induced severe or life‐threatening cytokine release syndrome. *Oncologist* **23**, 943–947 (2018). 10.1634/theoncologist.2018-0028 PMC6156173 29622697

[56] Lee DW (2014). Current concepts in the diagnosis and management of cytokine release syndrome. Blood.

[57] Gust J (2017). Endothelial activation and blood-brain barrier disruption in neurotoxicity after adoptive immunotherapy with CD19 CAR-T Cells. Cancer Discov..

[58] Norelli M (2018). Monocyte-derived IL-1 and IL-6 are differentially required for cytokine-release syndrome and neurotoxicity due to CAR T cells. Nat. Med..

[59] Zhang C, Wu Z, Li JW, Zhao H, Wang GQ (2020). The cytokine release syndrome (CRS) of severe COVID-19 and Interleukin-6 receptor (IL-6R) antagonist Tocilizumab may be the key to reduce the mortality. Int. J. Antimicrob. Agents.

[60] Troyer EA, Kohn JN, Hong S (2020). Are we facing a crashing wave of neuropsychiatric sequelae of COVID-19? Neuropsychiatric symptoms and potential immunologic mechanisms. Brain Behav. Immun..

