# Supplementary material for: The IL-6 antagonist tocilizumab is associated with worse depression and related symptoms in the medically ill
Source: Transl Psychiatry. 2021 Jan 18;11:58. doi: 10.1038/s41398-020-01164-y (PMC7812704; doi:10.1038/s41398-020-01164-y)
Supplement: Supplementary file 1 — Supplemental Tables for Disease-Restricted Subset Analysis [file 41398_2020_1164_MOESM1_ESM.docx]

**Supplemental Tables for Disease-Restricted Subset Analysis**

**Table 1.** Disease-Restricted Subset Analysis: Demographic and Clinical Characteristics

|  | Participants, No. (%) | | |
| --- | --- | --- | --- |
| **Characteristic** | **Tocilizumab Cohort**  **(N=24)** | **Control Cohort**  **(N=29)** | ***P-value*** |
| Age, mean (SD), y | 60.7 (10.6) | 54.0 (9.7) | 0.02 |
| Female sex | 10 (42) | 9 (31) | 0.42 |
| Race |  |  |  |
| Caucasian/White | 22 (92) | 28 (97) | 0.35 |
| Asian American | 1 (4) | 0 (0) |  |
| Latino/a | 1 (4) | 0 (0) |  |
| Native American | 0 (0) | 1 (3) |  |
| Body mass index, mean (SD) | 28.6 (4.7) | 29.3 (4.8) | 0.58 |
| Education^a,b^ |  |  |  |
| Less than 12 years | 1 (4) | 1 (4) | 0.86 |
| High school | 8 (35) | 10 (36) |  |
| Some college | 5 (22) | 9 (32) |  |
| College graduate | 4 (17) | 4 (14) |  |
| Post graduate degree | 4 (17) | 2 (7) |  |
| Trade school | 1 (4) | 2 (7) |  |
| Income^a,c^ |  |  |  |
| <$10,000 | 0 (0) | 0 (0) | 0.77 |
| $10,001-$25,000 | 4 (18) | 4 (14) |  |
| $25,001-$40,000 | 4 (18) | 5 (18) |  |
| $40,001-$55,000 | 4 (18) | 3 (11) |  |
| $55,001-$70,000 | 3 (14) | 4 (14) |  |
| $70,001-$85,000 | 3 (14) | 3 (11) |  |
| $85,001-$100,000 | 1 (5) | 6 (21) |  |
| >$100,000 | 3 (14) | 3 (11) |  |
| Diagnostic Category |  |  |  |
| ALL | 2 (8) | 2 (7) | 0.21 |
| AML | 15 (63) | 17 (59) |  |
| CML | 5 (21) | 2 (7) |  |
| MDS | 2 (8) | 8 (28) |  |
| Donor Type |  |  |  |
| 8/8 HLA matched unrelated donor | 14 (58) | 11 (38) | 0.14 |
| HLA matched sibling donor | 10 (42) | 18 (62) |  |
| Graft Type^d^ |  |  |  |
| Bone marrow | 5 (21) | 3 (11) | 0.34 |
| Mobilized peripheral blood stem cells | 19 (79) | 24 (89) |  |
| CMV positive | 8 (33) | 16 (55) | 0.11 |
| Conditioning Regimen |  |  |  |
| Myeloablative | 11 (46) | 26 (90) | <.001 |
| Reduced intensity conditioning | 13 (54) | 3 (10) |  |
| Development of aGVHD (Grade II-IV) |  |  |  |
| Baseline | 0 (0) | 0 (0) |  |
| Day 28 | 0 (0) | 2 (7) | 0.19 |
| Day 100 | 2 (8) | 7 (24) | 0.13 |
| Day 180 | 3 (13) | 10 (35) | 0.06 |

Abbreviations: ALL, acute lymphoblastic leukemia; AML, acute myelogenous leukemia; CLL, chronic lymphocytic leukemia; CML, chronic myelogenous leukemia; CMV, cytomegalovirus; HLA, human leukocyte antigen; MDS, myelodysplasia

^a^Data missing for one participant in the control cohort

^b^Data missing for one participant in the tocilizumab cohort

^c^Data missing for two participants in the tocilizumab cohort

^d^Data missing for two participants in the control cohort

**Table 2.** Disease-Restricted Subset Analysis: Quality of life patient reported outcomes at baseline, day 28, day 100, and day 180 post-transplant

|  | All (N=53) | | Tocilizumab Cohort (N=24) | | Control Cohort (N=29) | |  |
| --- | --- | --- | --- | --- | --- | --- | --- |
| Assessment | No. of Participants | Assessment Data, Mean Score (SD) | No. of Participants | Assessment Data, Mean Score (SD) | No. of Participants | Assessment Data, Mean Score (SD) | P-value |
| Depression |  |  |  |  |  |  |  |
| Baseline | 51 | 36.8 (11.3) | 24 | 34.5 (8.9) | 27 | 38.9 (13.0) | 0.38 |
| Day 28 | 47 | 41.2 (11.2) | 21 | 42.3 (10.3) | 26 | 40.3 (12.1) | 0.26 |
| Day 100 | 46 | 40.0 (12.1) | 20 | 39.5 (9.1) | 26 | 40.4 (14.1) | 0.63 |
| Day 180 | 33 | 38.8 (12.1) | 12 | 38.6 (9.9) | 21 | 38.9 (13.4) | 0.76 |
| Anxiety |  |  |  |  |  |  |  |
| Baseline | 51 | 15.7 (4.8) | 24 | 16.0 (5.1) | 27 | 15.6 (4.5) | 0.99 |
| Day 28 | 47 | 17.2 (4.5) | 21 | 17.5 (4.3) | 26 | 16.9 (4.6) | 0.48 |
| Day 100 | 45 | 18.2 (5.1) | 19 | 19.2 (6.0) | 26 | 17.4 (4.2) | 0.35 |
| Day 180 | 33 | 17.4 (5.4) | 12 | 18.2 (6.6) | 21 | 16.9 (4.7) | 0.96 |
| Fatigue, intensity |  |  |  |  |  |  |  |
| Baseline | 52 | 3.3 (2.0) | 24 | 2.4 (1.5) | 28 | 4.1 (2.1) | 0.001 |
| Day 28 | 47 | 4.4 (1.7) | 21 | 4.2 (1.8) | 26 | 4.5 (1.6) | 0.75 |
| Day 100 | 45 | 4.0 (1.9) | 18 | 3.7 (2.0) | 27 | 4.3 (1.9) | 0.28 |
| Day 180 | 33 | 4.2 (1.4) | 12 | 4.2 (1.1) | 21 | 4.2 (1.6) | 0.82 |
| Fatigue, duration |  |  |  |  |  |  |  |
| Baseline | 52 | 6.5 (4.2) | 24 | 5.1 (3.8) | 28 | 7.7 (4.2) | 0.04 |
| Day 28 | 47 | 9.6 (4.2) | 21 | 9.5 (4.8) | 26 | 9.7 (3.8) | 0.85 |
| Day 100 | 46 | 9.1 (4.5) | 19 | 8.9 (4.4) | 27 | 9.3 (4.6) | 0.73 |
| Day 180 | 33 | 9.4 (3.9) | 12 | 9.2 (4.0) | 21 | 9.6 (3.9) | 0.57 |
| Fatigue, interference |  |  |  |  |  |  |  |
| Baseline | 52 | 2.3 (2.3) | 24 | 1.5 (1.8) | 28 | 3.0 (2.6) | 0.02 |
| Day 28 | 47 | 3.2 (2.2) | 21 | 2.9 (2.0) | 26 | 3.4 (2.4) | 0.53 |
| Day 100 | 46 | 2.9 (2.4) | 19 | 2.3 (1.8) | 27 | 3.4 (2.7) | 0.17 |
| Day 180 | 33 | 2.8 (2.1) | 12 | 1.9 (1.7) | 21 | 3.2 (2.3) | 0.10 |
| Pain, intensity |  |  |  |  |  |  |  |
| Baseline | 52 | 1.7 (1.9) | 24 | 1.5 (1.7) | 28 | 1.8 (2.1) | 0.95 |
| Day 28 | 47 | 2.4 (2.1) | 21 | 3.1 (1.9) | 26 | 1.9 (2.1) | 0.03 |
| Day 100 | 45 | 1.9 (1.8) | 18 | 1.8 (1.8) | 27 | 2.0 (1.8) | 0.68 |
| Day 180 | 33 | 2.2 (2.2) | 12 | 2.2 (2.6) | 21 | 2.2 (2.0) | 0.79 |
| Pain, interference |  |  |  |  |  |  |  |
| Baseline | 52 | 1.3 (1.9) | 24 | 1.0 (1.7) | 28 | 1.6 (1.9) | 0.10 |
| Day 28 | 47 | 1.9 (2.1) | 21 | 2.0 (2.0) | 26 | 1.7 (2.2) | 0.32 |
| Day 100 | 45 | 2.0 (2.4) | 18 | 1.6 (1.8) | 27 | 2.2 (2.7) | 0.63 |
| Day 180 | 33 | 2.1 (2.4) | 12 | 1.6 (2.4) | 21 | 2.3 (2.4) | 0.30 |
| Sleep |  |  |  |  |  |  |  |
| Baseline | 52 | 7.2 (4.0) | 24 | 7.5 (4.2) | 28 | 6.9 (3.9) | 0.54 |
| Day 28 | 47 | 8.0 (4.5) | 21 | 8.1 (4.4) | 26 | 7.8 (4.7) | 0.61 |
| Day 100 | 47 | 7.2 (4.0) | 20 | 7.9 (4.5) | 27 | 6.7 (3.6) | 0.42 |
| Day 180 | 33 | 7.7 (4.0) | 12 | 8.5 (4.0) | 21 | 7.2 (3.9) | 0.38 |

**Table 3.** Disease-Restricted Subset Analysis: Tocilizumab compared to control cohort based on propensity-weighted^a^ models for quality of life patient-reported outcomes after adjusting for baseline values and aGVHD (grade II-IV)

|  | **Day 28** | | **Day 100** | | **Day 180** | |
| --- | --- | --- | --- | --- | --- | --- |
| **Assessment** | **Estimate (95% CI)** | **P-value** | **Estimate (95% CI)** | **P-value** | **Estimate (95% CI)** | **P-value** |
| Depression | 4.25 (-1.36, 9.86) | 0.14 | 0.40 (-5.38, 6.18) | 0.89 | 3.74 (-2.96, 10.44) | 0.27 |
| Anxiety | 0.04 (-2.42, 2.51) | 0.97 | 0.77 (-1.79, 3.34) | 0.55 | 0.44 (-2.51, 3.40) | 0.77 |
| Fatigue, intensity | -0.22 (-1.29, 0.85) | 0.69 | -0.74 (-1.83, 0.35) | 0.18 | 0.20 (-1.03, 1.42) | 0.75 |
| Fatigue, duration | 0.44 (-1.91, 2.79) | 0.71 | 0.36 (-2.04, 2.77) | 0.77 | 0.14 (-2.63, 2.90) | 0.92 |
| Fatigue, interference | -0.46 (-1.64, 0.72) | 0.44 | -0.77 (-1.97, 0.44) | 0.21 | -0.87 (-2.25, 0.52) | 0.22 |
| Pain, intensity | 1.06 (-0.03, 2.15) | 0.06 | 0.00 (-1.13, 1.13) | 1.00 | 0.79 (-0.50, 2.08) | 0.23 |
| Pain, interference | 0.22 (-0.96, 1.41) | 0.71 | -0.50 (-1.73, 0.73) | 0.43 | -0.15 (-1.57, 1.26) | 0.83 |
| Sleep | -0.30 (-2.50, 1.90) | 0.77 | 0.96 (-1.28, 3.19) | 0.40 | 1.67 (-0.89, 4.23) | 0.20 |

^a^Propensity scores were obtained using a logistic regression model with age, sex, BMI, CMV status, conditioning regimen, and donor type as predictors of study group.

**Table 4:** Disease-Restricted Subset Analysis: Effect of presence of aGVHD (grade II-IV) compared to those without aGVHD at same time point, based on propensity-weighted models for quality of life patient-reported outcomes after adjusting for baseline values and cohort

| **Assessment** | **Estimate (95% CI)** | **P-value** |
| --- | --- | --- |
| Depression | 2.12 (-5.04, 9.28) | 0.50 |
| Anxiety | -0.09 (-3.07, 2.89) | 0.95 |
| Fatigue, intensity | 0.32 (-1.05, 1.69) | 0.59 |
| Fatigue, duration | 1.81 (-1.24, 4.87) | 0.20 |
| Fatigue, interference | 0.73 (-0.80, 2.25) | 0.29 |
| Pain, intensity | -0.06 (-1.50, 1.38) | 0.92 |
| Pain, interference | -0.34 (-1.82, 1.15) | 0.60 |
| Sleep | 0.06 (-2.75, 2.88) | 0.96 |

^a^Propensity scores were obtained using a logistic regression model with age, sex, BMI, CMV status, conditioning regimen, and donor type as predictors of study group.
